# Comparative Genomic Analyses of Cellulolytic Machinery Reveal Two Nutritional Strategies of Marine Labyrinthulomycetes Protists

**DOI:** 10.1128/spectrum.04247-22

**Published:** 2023-02-06

**Authors:** Xiuping Liu, Lu Lyu, Jiaqian Li, Biswarup Sen, Mohan Bai, Jason E. Stajich, Jackie L. Collier, Guangyi Wang

**Affiliations:** a Center for Marine Environmental Ecology, School of Environmental Science and Engineering, Tianjin University, Tianjin, China; b Frontiers Science Center for Synthetic Biology, Tianjin University, Tianjin, China; c Key Laboratory of Systems Bioengineering (MOE), Tianjin University, Tianjin, China; d Center for Biosafety Research and Strategy, Tianjin University, Tianjin, China; e Department of Plant Pathology and Microbiology, University of California, Riverside, California, USA; f School of Marine and Atmospheric Sciences, Stony Brook University, Stony Brook, New York, USA; Center of Innovative and Applied Bioprocessing

**Keywords:** Labyrinthulomycetes, cellulase, ecological function, comparative genomics, carbon cycling

## Abstract

Labyrinthulomycetes are a group of ubiquitous and diverse unicellular Stramenopiles and have long been known for their vital role in ocean carbon cycling. However, their ecological function from the perspective of organic matter degradation remains poorly understood. This study reports high-quality genomes of two newly isolated Labyrinthulomycetes strains, namely, *Botryochytrium* sp. strain S-28 and *Oblongichytrium* sp. strain S-429, and provides molecular analysis of their ecological functions using comparative genomics and a biochemical assay. Our results suggest that Labyrinthulomycetes may occupy multiple ecological niches in marine ecosystems because of the significant differences in gene function among different genera. Certain strains could degrade wheat bran independently by secreting cellulase. The key glycoside hydrolase families (GH1, GH5, and GH9) related to cellulase and the functional domains of carbohydrate-active enzymes (CAZymes) were more enriched in their genomes. This group can actively participate in marine biochemical cycles as decomposers. In contrast, other strains that could not produce cellulase may thrive as “leftover scavengers” and act as a source of nutrients to the higher-trophic-level plankton. In addition, our findings emphasize the dual roles of endoglucanase, acting as both exo- and endoglucanases, in the process of cellulose degradation. Using genomic, biochemical, and phylogenetic analyses, our study provides a broader insight into the nutritional patterns and ecological functions of Labyrinthulomycetes.

**IMPORTANCE** Unicellular heterotrophic eukaryotes are an important component of marine ecosystems. However, their ecological functions and modes of nutrition remain largely unknown. Our current understanding of marine microbial ecology is incomplete without integrating these heterotrophic microeukaryotes into the food web models. This study focuses on the unicellular fungus-like protists Labyrinthulomycetes and provides two high-quality genomes of cellulase-producing Labyrinthulomycetes. Our study uncovers the basis of their cellulase production by deciphering the results of genomic, biochemical, and phylogenetic analyses. This study instigates a further investigation of the molecular mechanism of organic matter utilization by Labyrinthulomycetes in the world’s oceans.

## INTRODUCTION

Labyrinthulomycetes are unicellular heterotrophic eukaryotes with a ubiquitous presence in the world’s oceans (1–3). Their biomass has been reported to approach or exceed that of planktonic prokaryotes in coastal and oceanic waters (4, 5). Recent findings of their dominant presence in the pelagic waters, marine snow, and sediments in different ocean provinces suggest that they potentially play a key role in transporting particulate organic matter (POM) from the surface to the dark ocean in the biological carbon pump (BCP) (1, 6, 7). Their vertical distribution patterns in pelagic waters revealed that they partitioned into different phylotype groups based on depth (7). Particularly, the high-resolution time-series observations indicated that the majority of Labyrinthulomycetes phylotypes display short and repetitive season-specific blooms, and some of them exhibit time-lagged correlations or cooccurrences with bacterial, algal, or fungal phylotypes (3). Thus, Labyrinthulomycetes likely drive the carbon and nutrient cycling through multifaceted nutritional models in the world’s oceans.

As of now, our understanding of the nutritional lifestyles of Labyrinthulomycetes is largely based on laboratory studies of culturable thraustochytrids (8, 9). They are widely distributed in estuarine waters, sediments, and mangroves (8, 10–14). Their cells could penetrate organic particles and secrete degradative enzymes to digest organic material via ectoplasmic nets, thereby achieving the absorption of nutrients (13, 15). Previous studies have found that thraustochytrids utilize various types of marine organic substrates by secreting extracellular hydrolytic enzymes (e.g., amylases, cellulases, lipases, proteases, phosphatases, pectinases, and xylanases) (16, 17). High densities of thraustochytrids have been observed in algal, mangrove, and seagrass detritus (18–21). During the period of detrital decomposition, dissolved organic matter (DOM) rapidly leaches out (18, 21). In addition, thraustochytrid abundance was found to significantly correlate with the particulate organic carbon (POC) concentration. The close coupling of thraustochytrids and POC implied certain functional activity of thraustochytrids (e.g., exoenzymatic degradation) on POM (22). Thus, they have been considered one of the major mineralizers and decomposers in marine ecosystems, with a presumably significant role in ocean carbon cycling.

Of the hydrolases derived from thraustochytrids, cellulases have been considered the most important enzymes in the degradation of plant or phytoplankton detritus due to their function of disrupting plant cell walls (16, 23). The cellulase system constitutes endoglucanase, exoglucanase, and β-glucosidase, and thus depolymerization of cellulosic substrates requires their synergistic action (see Fig. S1 in the supplemental material) (24, 25). Strains belonging to *Aplanochytrium*, *Botryochytrium*, *Oblongichytrium*, *Parietichytrium*, *Schizochytrium*, *Sicyoidochytrium*, *Thraustochytrium*, and *Ulkenia* genera have been reported to produce cellulases (16, 26). Furthermore, studies have also revealed that extracellular enzyme production is strain specific (27). For example, *Aurantiochytrium* sp. strain KRS101 could utilize molasses and carboxymethyl cellulose (CMC) for its growth (28), whereas two polyunsaturated fatty acid (PUFA)-producing *Aurantiochytrium* strains, namely, Mn4 and SW8, lacked complete machinery of hydrolytic enzymes for degrading plant-derived organic materials (29). The latter strains were considered to play an important role as a nutritional source rather than as a decomposer in the marine microbial food web. Interestingly, two nutritional strategies of thraustochytrids had been speculated to exist in the world’s oceans. The ecotype that produces PUFAs serves as a dietary source for the plankton of higher trophic levels and likely lives as a “leftover scavenger” of bacterioplankton, whereas the other ecotype produces cellulosic enzymes and thrives on detrital marine organic matter (4, 9, 29, 30). Nevertheless, our understanding of their nutritional model at the molecular level remains largely elusive.

*De novo* genome assembly enables functional genomics of species, and comparative genomics can provide new insight into the molecular machinery of organisms (29, 31). For example, comparative genomics revealed the host distribution and predicted molecular and metabolic properties of *Thaumarchaeota*, suggesting their various degrees of specialization in the sponge environment (32). *Aquimarina* carries multiple genes for the degradation of marine carbohydrates and vitamin biosynthesis (33). These genes might promote nutrient exchange between strains and the algal host, thereby revealing their ecological role in the marine environment. Currently, the genomic studies on Labyrinthulomycetes mostly focus on the PUFA-producing strains to reveal the mechanisms of PUFA synthesis. Genomic studies on the mechanism of biodegradation by Labyrinthulomycetes are sparse (31, 34–36).

In this study, we report two high-quality genomes of cellulase-producing Labyrinthulomycetes: *Botryochytrium* sp. strain S-28 and *Oblongichytrium* sp. strain S-429. Our study is the first report on the molecular basis of cellulase production and the differences in molecular machinery between cellulase-producing and non-cellulase-producing Labyrinthulomycete strains. The results provide a basis for future research on the molecular mechanisms of organic matter utilization by Labyrinthulomycetes.

## RESULTS

### Genomic features.

The genomes of *Botryochytrium* sp. strain S-28 (S-28) and *Oblongichytrium* sp. strain S-429 (S-429) were sequenced on the Illumina NovaSeq and Oxford Nanopore PromethION sequencers and assembled (see Table S1 in the supplemental material). The GC contents of the hybrid assemblies of S-28 and S-429 were 47.47 mol% and 43.30 mol%, respectively. The hybrid assemblies yielded total lengths of 38,142,407 bp (44 contigs) and 44,355,669 bp (45 contigs) for S-28 and S-429, respectively. The estimated genome size of S-28 was 36.22 Mb with an *N*_50_ value of 869,909 bp (NextDenovo output), whereas the genome size of S-429 was 43.24 Mb with an *N*_50_ of 1,366,673 bp (NECAT output). The maximum contig lengths of S-28 and S-429 were 2,173,176 and 2,291,525, respectively, and the minimum contig lengths were 196,210 and 78,501, respectively. The genome sizes of S-28 and S-429 were significantly smaller than those of Mn4 (65.69 Mb) (29), SW8 (61.67 Mb) (29), or other thraustochytrids (about 60 Mb) (31, 34). This may be due to less redundancy in the genomes of S-28 and S-429. The integrity of genome assembly was assessed using BUSCO (37), which revealed good completeness of haplotypes (S-28, 89.00%; S-429, 81.00%) of both genomes. The levels of genomic completeness of Mn4 and SW8 were 91.4% and 91.8%, respectively. The genome sizes of S-28 and S-429 were similar to that of Schizochytrium aggregatum ATCC 28209 (38.96 Mb) (29). Although *Aurantiochytrium* sp. strain Mn4 (15,154) and *Aurantiochytrium* sp. strain SW8 (14,402) had larger genomes, the S-28 (18,696) and S-429 (18,058) genomes contained more protein-coding sequences or predicted genes (Table 1).

**TABLE 1:** Genomic features of S-28, S-429, Mn4, and SW8 strains of Labyrinthulomycetes

Feature	Result for strain:			
	S-28	S-429	Mn4	SW8
Scaffolds, no.	44	45	1,611	1,202
Genome size, Mb	36.22	43.24	65.69	61.67
Maximum length, bp	2,173,176	2,291,525	667,038	659,658
*N*_50_, bp	869,909	1,366,673	153,854	127,831
*N*_90_, bp	597,294	761,285	14,564	22,659
GC content, mol%	47.47	43.30	44.93	45.11
Rate of N, %	0.00	0.00	0.00	0.00
No. of predicted CDSs	18,696	18,058	15,154	14,402
No. of genes annotated with:				
COG database	5,828	5,995	5,295	5,229
KEGG database	3,391	3,698	1,817	1,839

### Phylogenetic and phylogenomic relationships.

Phylogenetic analysis using 18S rRNA gene sequences suggested that S-28 was closely related to the members of *Botryochytrium*, with a 99.08% DNA similarity to *Botryochytrium* sp. strain SEK 597 (Fig. S2). Conversely, S-429 clustered with the members of the *Oblongichytrium* genus, with a 99.66% similarity with *Oblongichytrium* sp. strain SEK 708. Based on these findings, we conclude that S-28 and S-429 have close kinship with the *Botryochytrium* and *Oblongichytrium* genera, respectively.

**FIG 1:**
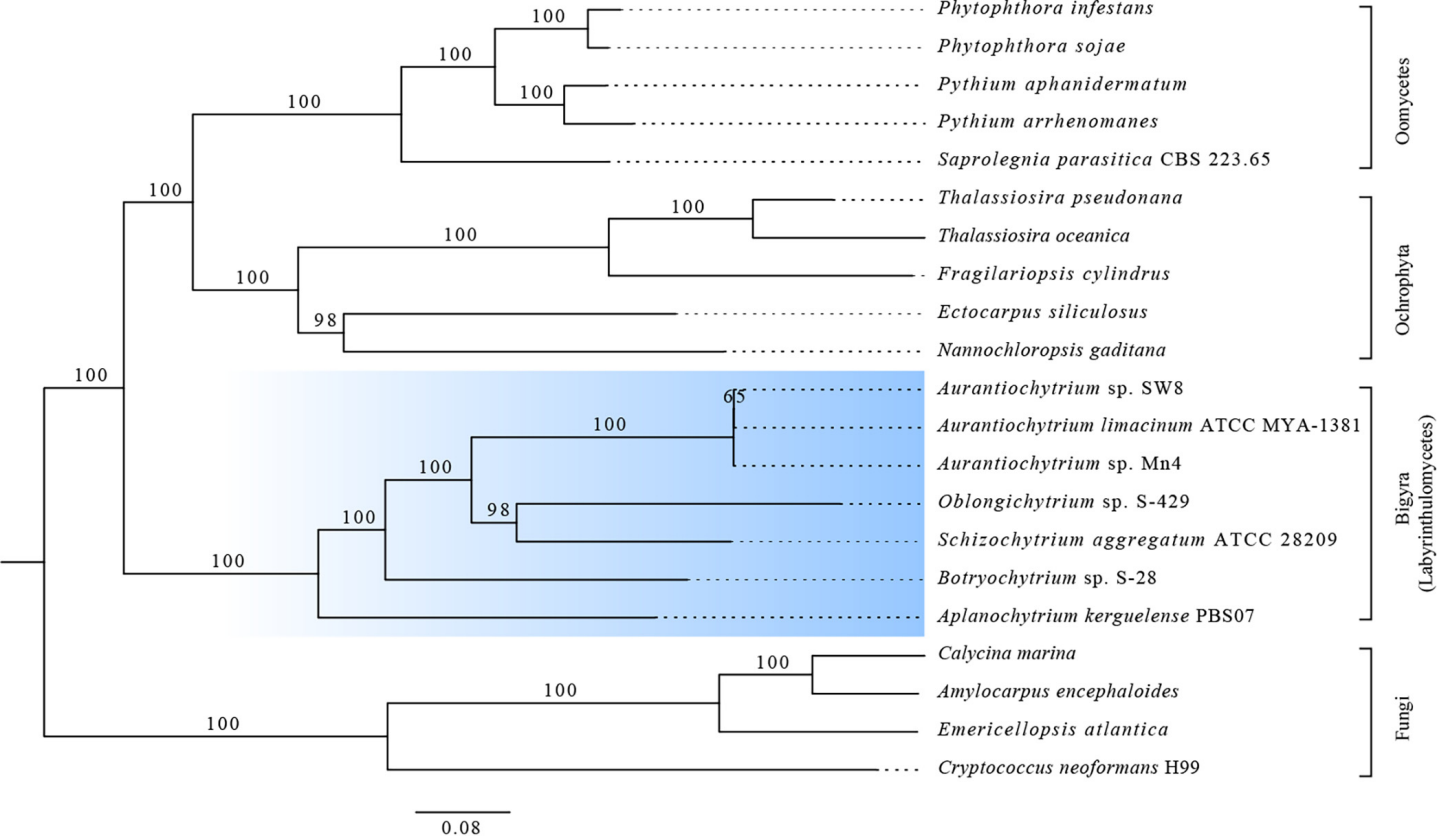
Phylogeny of eukaryotes based on orthologous gene groups demonstrating the branching position of Labyrinthulomycetes. Labyrinthulomycetes are highlighted in blue.

Furthermore, 21 eukaryotic species, including members of the Stramenopiles and Fungi, were selected to assess the phylogenetic relationship of the Labyrinthulomycete strains. Their amino acid sequences were downloaded from the JGI database, and orthogroups were generated using OrthoFinder. In total, 287,635 genes (88.0% of the total) were assigned to 35,499 orthogroups, of which 1,139 single-copy orthogroups were found in all 21 species. A phylogenetic tree was constructed from concatenated alignments of these single-copy genes (Fig. 1). The resulting tree topology indicated that S-28 and S-429 formed a sister branch to the Labyrinthulomycetes radiation. Within the Stramenopiles, Oomycetes and the photosynthetic Stramenopiles (Ochrophyta) formed a monophyletic sibling clade to Bigyra (e.g., Labyrinthulomycetes), which was somewhat consistent with previously published phylogenetic analyses (38, 39). Furthermore, one interpretation of the multilocus phylogenies suggested that the Labyrinthulomycetes stem lineage replaced phagotrophy with absorptive nutrition through an ectoplasmic network early in its evolution (38). As shown in the phylogenetic tree (Fig. 1), Mn4, SW8, and *A. limacinum* ATCC MYA-1381 cluster as sister lineages and belong to the *Aurantiochytrium* genus. Thus, Mn4 and SW8 were slightly distant from S-28 and S-429, while other strains of different genera, such as Schizochytrium aggregatum ATCC 28209 and Aplanochytrium kerguelense PBS07, formed different clades, which was consistent with the classification results of the 18S rRNA phylogenetic tree (Fig. S2). These results suggest that they diverged during evolution.

### Comparative cellulolytic activities.

**FIG 2:**
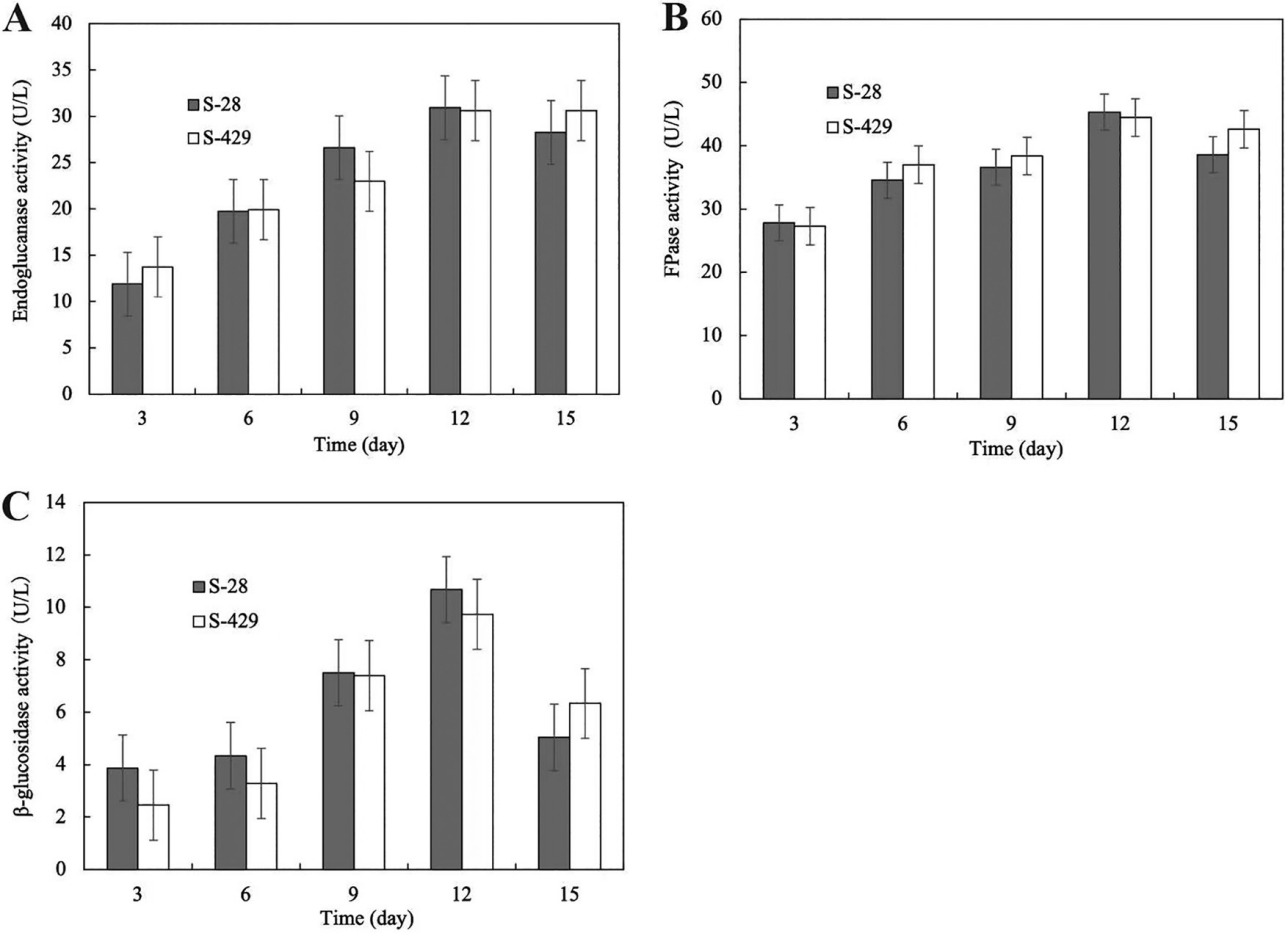
Cellulase activities of S-28 and S-429. (A to C) Production of (A) endoglucanase, (B) FPase, and (C) β-glucosidase by S-28 and S-429 grown on wheat bran. Enzyme activities were determined at intervals of 3 days. All analyses were performed in triplicate, and standard deviations are indicated by error bars.

The cellulolytic activities of the S-28 and S-429 strains were compared with that of the Mn4 strain by qualitative and quantitative analyses. Distinct clear transparent zones were observed around the colonies of S-28 and S-429 but not of Mn4 (Fig. S3). Furthermore, both S-28 and S-429 exhibited increased activities of endoglucanase, filter paper cellulase (FPase), and β-glucosidase upon prolonged cultivation (Fig. 2). In contrast, no reducing sugars were detected in the Mn4 culture broth. The cellulase activities of S-28 and S-429 peaked on day 12 of cultivation. The endoglucanase (Fig. 2A), FPase (Fig. 2B), and β-glucosidase (Fig. 2C) activities of S-28 reached 30.9, 45.3, and 10.7 U/L, respectively. Likewise, these activities of S-429 reached 30.6, 44.4, and 9.7 U/L, respectively. These results indicate that both S-28 and S-429 can break down and utilize wheat bran by secreting cellulase.

Although the genes encoding exoglucanase could not be identified in the S-28 and S-429 genomes, the FPase activity was detected in both strains, suggesting their complete extracellular cellulase activities. These findings further indicate that other enzymes must occur in their genomes to compensate for the exoglucanase activity. Taken together, our study provides evidence for two different nutritional strategies of Labyrinthulomycetes.

### Functional divergence.

This study examined differences in the protein-coding gene content of cellulase-producing (S-28 and S-429) and non-cellulase-producing (Mn4 and SW8) Labyrinthulomycetes strains. The four strains shared 5,309 core orthogroups, which accounted for 31.64 to 39.95% of the total gene content in individual genomes (Fig. S4). Among these core orthogroups, hydrolase activity, ion binding, and transferase activity were identified as common molecular functions (Table S2). Each strain had lineage-specific gene content as well. S-28, S-429, Mn4, and SW8 had 894, 1,297, 122, and 42 unique orthologous groups, respectively (Fig. S4). Furthermore, S-28 and S-429 shared 487 orthogroups not present in Mn4 or SW8 (Fig. S4), in which a total of 1,728 proteins were identified. These proteins have molecular functions in oxidoreductase activity, transferase activity, and hydrolase activity (Table S2).

**FIG 3:**
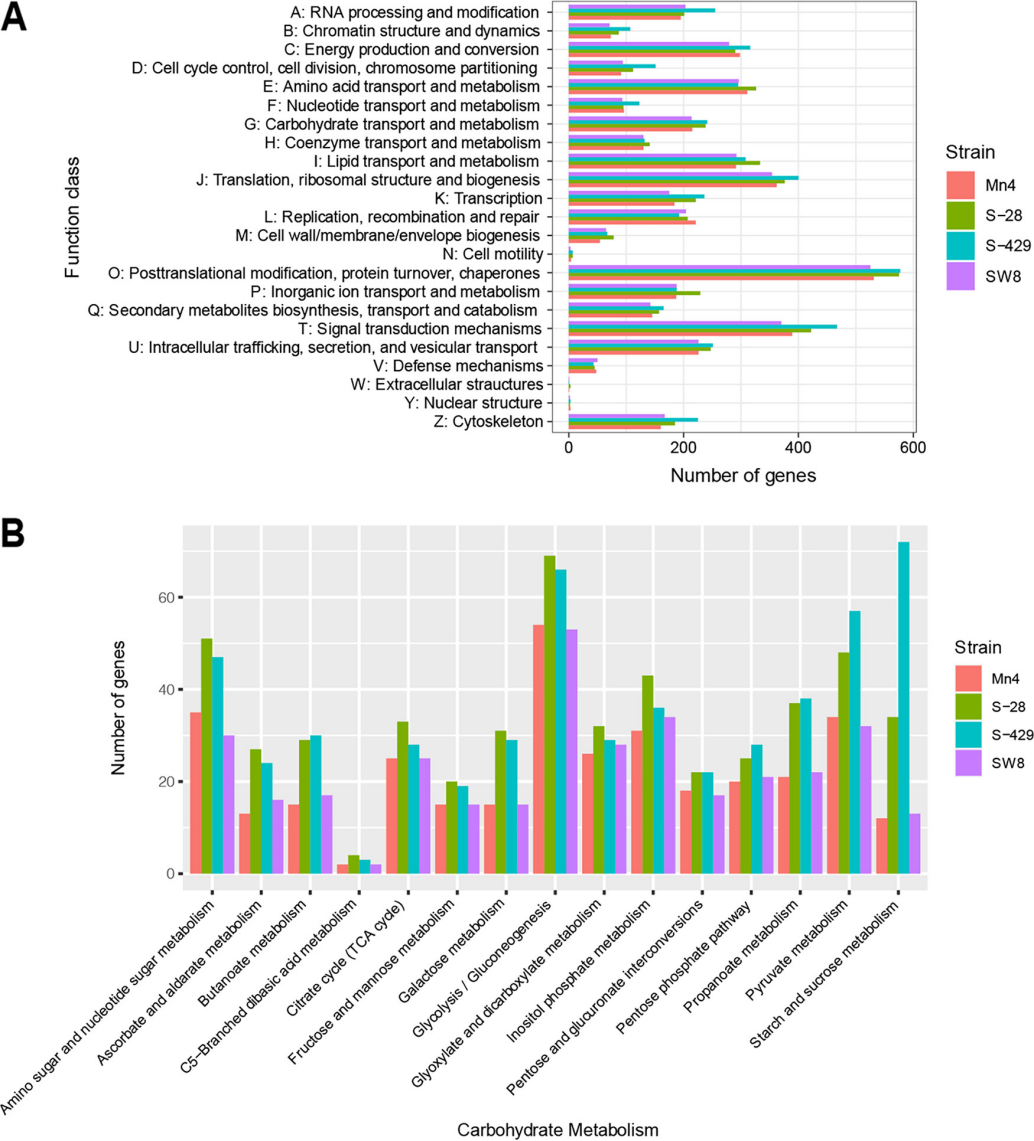
Gene function annotation of *Botryochytrium* sp. strain S-28, *Oblongichytrium* sp. strain S-429, *Aurantiochytrium* sp. strain Mn4, and *Aurantiochytrium* sp. strain SW8. (A) Comparison of functional categories of genes annotated by the COG database and (B) secondary function classification for the number of genes in the KEGG carbohydrate metabolism pathway.

To further understand the differences in the gene functions between cellulase-producing and non-cellulase-producing groups, COG and KEGG pathway analyses were performed. All four strains shared similar COG assignment profiles (Fig. 3A and Table S3), and consistent with the greater number of total coding DNA sequences (CDSs), S-28 and S-429 often had more genes in each category than Mn4 and SW8 (Fig. 3A). The most abundant categories among these strains were O (posttranslational modification, protein turnover, and chaperones [9.62 to 10.04%]) and T (signal transduction mechanisms [7.08 to 7.79%]). Further functional annotation using the KEGG database identified 3,391 and 3,698 KEGG pathway genes in S-28 and S-429, respectively. In addition, differences in the 15 KEGG pathways involved in carbohydrate metabolism among the four strains were revealed (Fig. 3B). Thirty-four genes in S-28 and 72 genes in S-429 were found to be associated with sucrose and starch metabolism. In contrast, the genomes of Mn4 and SW8 contained fewer genes (i.e., 12 and 13 genes, respectively) involved in sucrose and starch metabolism. These findings suggest that S-28 and S-429 may have competency for the utilization of sucrose and starch.

### Comparison of the proportions of eukaryotic CAZymes.

**FIG 4:**
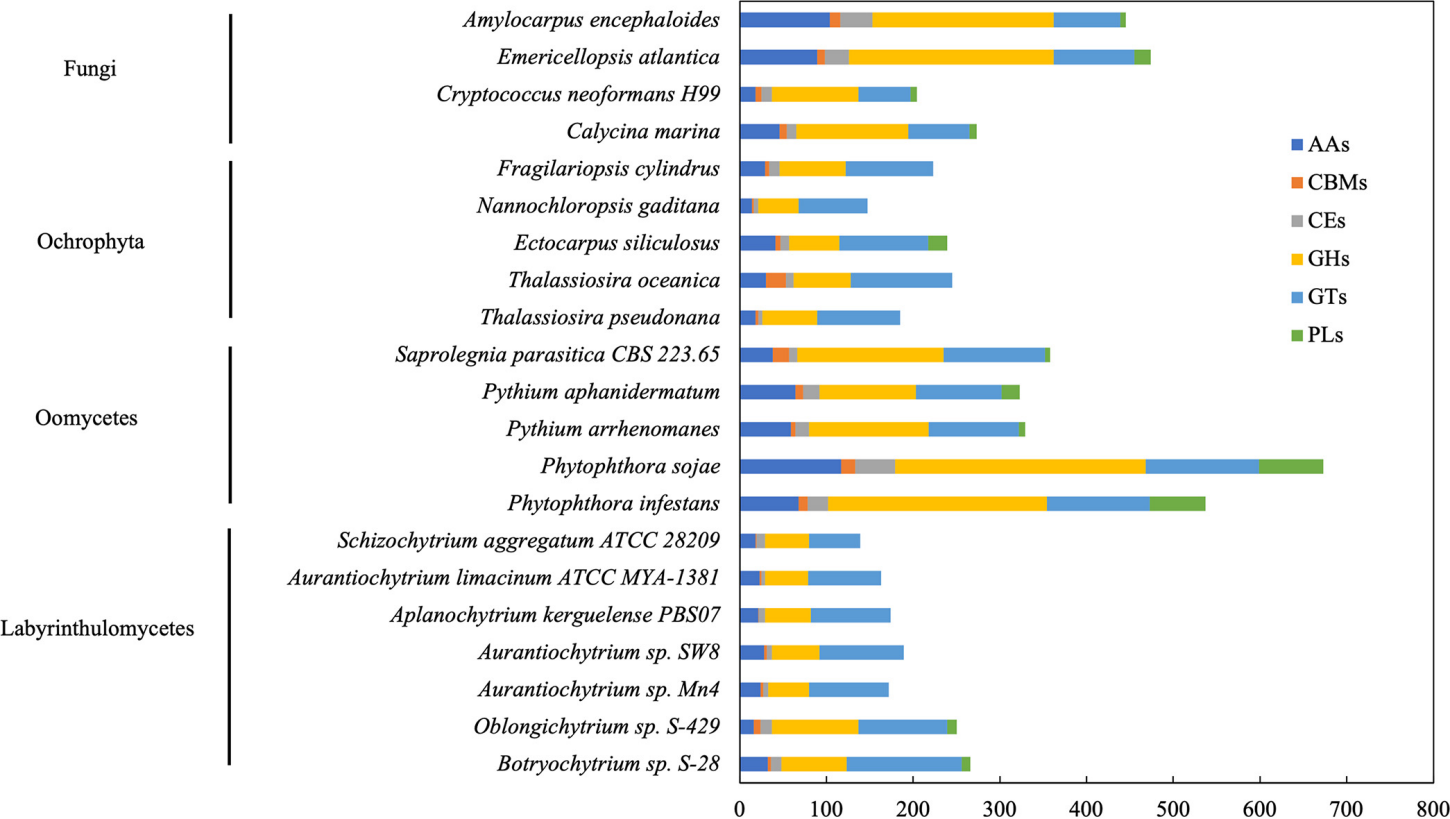
Comparison of the distribution of CAZyme families in Fungi, Ochrophyta, Oomycetes, and Labyrinthulomycetes.

The genes associated with carbohydrate-active enzymes (CAZymes) were identified from the predicted gene sets of a total of 21 eukaryote species (Fig. 4). Fungi (2.86 to 4.76%) and Oomycetes (1.78 to 3.02%) had greater proportions of genes (or sequences) encoding CAZymes than Ochrophyta (0.71 to 1.62%) and Labyrinthulomycetes (1.10 to 1.46%) (Table S4). Furthermore, all of the six CAZyme families, namely, carbohydrate esterases (CEs), glycoside hydrolases (GHs), glycosyltransferases (GTs), polysaccharide lyases (PLs), carbohydrate-binding modules (CBMs), and enzymes performing auxiliary activities (AAs) (40, 41), were present in all of the species of Fungi and Oomycetes (Fig. 4), consistent with the plant-associated ecological functions of these organisms.

Most CAZymes in Labyrinthulomycetes were members of the GT (40.8 to 53.5%) and GH (27.3 to 40.0%) families, which include known cellulose-degrading enzymes (Table 2). However, except in S-28 and S-429, the PLs were absent in other Labyrinthulomycetes, which suggested that these two strains are possibly better degraders of polysaccharides in the ocean. Furthermore, S-28 and S-429 showed the highest numbers of CAZyme-encoding genes among the Labyrinthulomycetes strains (Table S5), which indicated that these two strains may have the potential to degrade more different types of carbohydrates.

**TABLE 2:** Distribution of CAZyme families across different *Labyrinthulomycete* genomes

Family	No. (%) of CAZymes from family shown						
	*Botryochytrium* sp. strain S-28	*Oblongichytrium* sp. strain S-429	*Aurantiochytrium* sp. strain Mn4	*Aurantiochytrium* sp. strain SW8	Aplanochytrium kerguelense PBS07	Aurantiochytrium limacinum ATCC MYA-1381	Schizochytrium aggregatum ATCC 28209
CEs	12 (4.51)	13 (5.20)	6 (3.49)	6 (3.17)	7 (4.02)	4 (2.45)	10 (7.19)
GHs	75 (28.20)	100 (40.00)	47 (27.33)	55 (29.10)	53 (30.46)	50 (30.67)	51 (36.69)
GTs	133 (50.00)	102 (40.80)	92 (53.49)	97 (51.32)	92 (52.87)	84 (51.53)	59 (42.45)
PLs	10 (3.76)	11 (4.40)	0	0	0	0	0
CBMs	4 (1.50)	8 (3.20)	3 (1.74)	3 (1.59)	1 (0.57)	2 (1.23)	1 (0.72)
AAs	32 (12.03)	16 (6.40)	24 (13.95)	28 (14.81)	21 (12.07)	23 (14.11)	18 (12.95)
Total	266	250	172	189	174	163	139

The CAZyme families that were present in all the seven Labyrinthulomycete genomes can be the ancestral gene family, with a total of 34 such CAZyme families. These ancestral gene families in the genomes of three *Aurantiochytrium* strains (49.28 to 51.52%) and one *Schizochytrium* strain (50.75%) accounted for a higher proportion of their CAZyme-encoding genes, followed by *Aplanochytrium* (44.74%). Notably, *Botryochytrium* and *Oblongichytrium* had the lowest proportions (36.96 to 40.48%) of the ancestral gene family (Table 2). Therefore, the sibship among non-cellulase-producing strains (strains belonging to *Aurantiochytrium*) was closer than that of strains belonging to another genus.

The degradation of organic matter by Labyrinthulomycetes is mainly achieved by secreting extracellular enzymes (Fig. S5). Therefore, whether the enzyme can be secreted out of the cell is a prerequisite for its biodegradation potential. In this study, the secretomes of the predicted CAZymes in some Labyrinthulomycete strains were identified. The total number of secreted CAZymes in their genomes ranged from 18 to 79 (Table S6). GHs accounted for the most CAZyme domains across all secretomes. Interestingly, S-28 and S-429 had 79 and 71 secreted CAZymes, respectively, which were much higher numbers than those of other strains. Furthermore, secreted PLs and CBMs were found only in S-28 and S-429. The absence of genes encoding key GH families (GH3, GH5_2 [GH5 subfamily 2], and GH9) involved in cellulose degradation was also observed in the secretomes of *Aplanochytrium* and *Aurantiochytrium* strains, which indicated that these strains may not be able to secrete cellulase. These findings support previous studies that reported the lack of cellulase activities in some *Aurantiochytrium* strains (16, 29). Interestingly, GH3, GH5_2, and GH9 genes were found in the secretomes of S-28 and S-429, suggesting their ability to produce extracellular cellulase, consistent with a previous study (16).

### Hydrolase domains.

**FIG 5:**
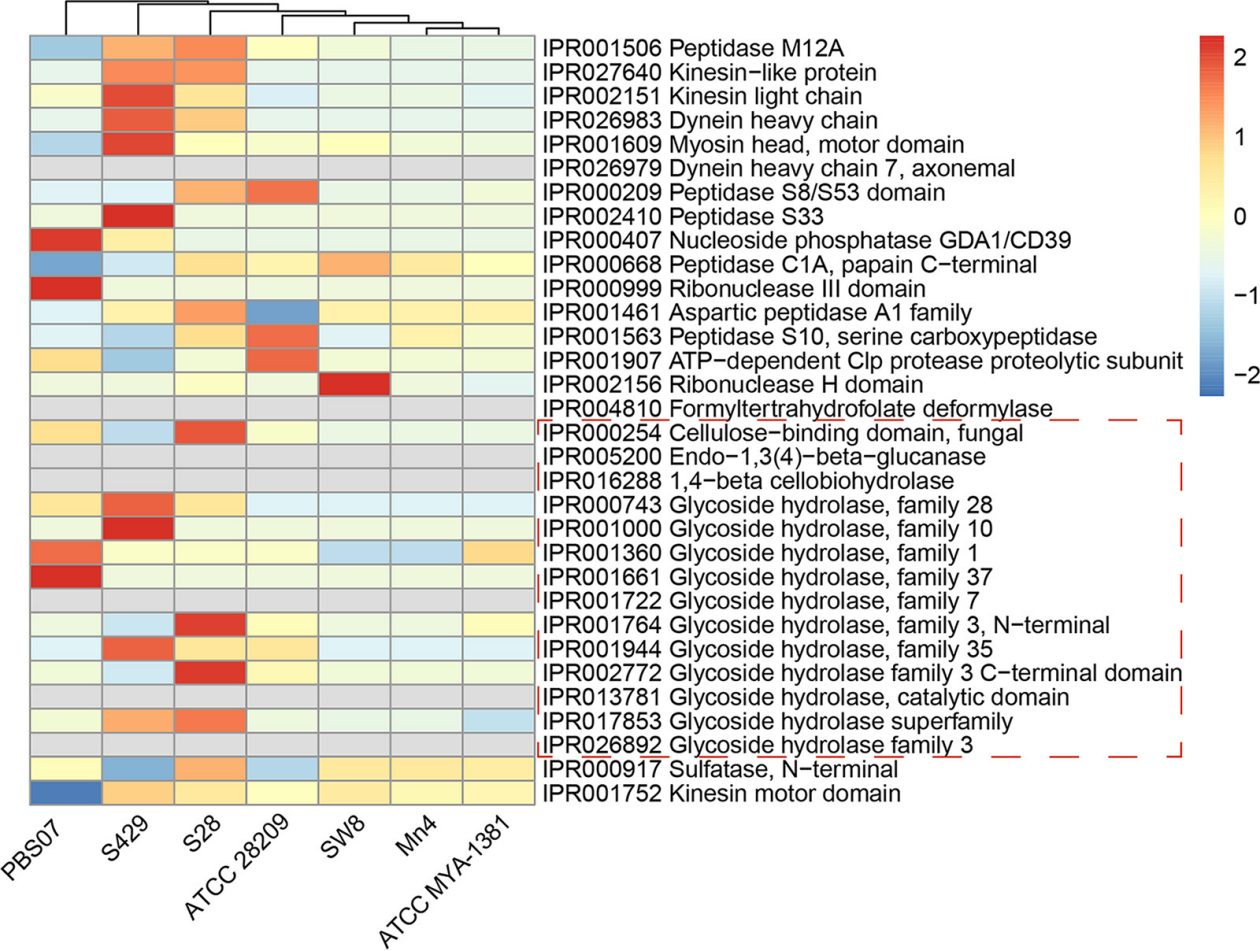
IPR domains of hydrolase activity enriched in S-28 and S-429 compared to other Labyrinthulomycete strains (*Aurantiochytrium* sp. strain Mn4, *Aurantiochytrium* sp. strain SW8, Aplanochytrium kerguelense PBS07, Schizochytrium aggregatum ATCC 28209, and Aurantiochytrium limacinum ATCC MYA-1381). Fourteen domains in carbohydrate metabolism with hydrolase activity are circled by a dashed line. All values were homogenized and colored along a color scale from blue (low) to red (high).

The domains associated with cellulases, namely, IPR005200 [endo-1,3(4)-β-glucanase] and IPR016288 (1,4-β-cellobiohydrolase), were absent in the genomes of all Labyrinthulomycetes strains (Fig. 5). The absence of these domains suggested the lack of a complete machinery of cellulase formation. However, the cellulose-binding domain (IPR000254) was annotated in the genome of S-28 but absent in S-429, which suggested that the latter strain seems to utilize other domains for cellulase activity. Furthermore, several domains (i.e., IPR000743, IPR001000, IPR001360, IPR001764, IPR001944, IPR002772, and IPR017853) classified as GH families were found in S-28 and S-429. These domains are known to decompose cellulose, hemicellulose, pectin, and trehalose—the major plant cell wall components (29). All domains related to CAZymes were less enriched in the *Aurantiochytrium* strains, namely, Mn4, SW8, and ATCC MYA-1381 (Fig. 5), which may be related to their different biological functions. *Aurantiochytrium* strains are mostly reported to produce docosahexaenoic acid (DHA) and not degrade polysaccharides.

Overall, our results suggest that the cellulase-producing and non-cellulase-producing strains were different in terms of their domains, especially those related to GHs involved in decomposing plant cell walls. Moreover, the non-cellulase-producing strains had fewer functional domains annotated to CAZymes and lower numbers of secreted CAZymes.

### GH families.

The identification of cellulases revealed 16 proteins in S-28 and 6 proteins in S-429, respectively (Table S6). These proteins were assigned to GH1, GH3, GH5 subfamily 2 (GH5_2), GH5 subfamily 5 (GH5_5), GH5 subfamily 12 (GH5_12), and the GH9 family. They are key GH families of endoglucanase and β-glucosidase for the breakdown of cellulose into glucose (42). On the other hand, only two putative β-glucosidase proteins belonging to the GH3 family were annotated and genes encoding endoglucanase and exoglucanase were absent in Mn4 and SW8 genomes. Additionally, the genes encoding the secreted proteins of GH1, GH5, and GH9 were also absent in Mn4 and SW8 secretomes.

The structures and active sites of GH5 and GH9 protein families (Thermomonospora fusca Cel5 [ThrCel5] and ThrCel9) were compared with those of two reported GH5 and GH9 protein families (Thermobifida halotolerans Cel5A [ThCel5A] and E4), respectively (Fig. S6). The protein structure model of ThrCel9 (Fig. S6A) was most similar to that of E4 (Fig. S6B), with a sequence similarity of 37.86%. Their structures contained a catalytic domain with an (α/α)_6_ barrel fold and a cellulosic binding domain with a β-sandwich fold, which helps the enzyme to act on cellotetraose (43). The catalytic domains of both ThrCel5 (Fig. S6C) and ThCel5A (Fig. S6D) folded as a (β/α)_8_ barrel. The orientation of the key residues in the active site of ThrCel5 was similar to that of ThCel5A, concerning amino acids Arg216, His256, His359, Glu294, and Glu386 in ThCel5A. These structural features suggest that endoglucanase from Labyrinthulomycetes may have the dual function of exoglucanase and endoglucanase.

Furthermore, we identified chitinases in secretomes of Labyrinthulomycetes (Table S6), which may be capable of degrading polysaccharides from marine crustacean shells. Glycoside hydrolases (GH43 and GH3) involved in the degradation of pectin, lignin, and xylan were also identified. The proteins encoding putative hemicellulase were found in all four Labyrinthulomycete genomes.

## DISCUSSION

Labyrinthulomycetes can have a significant impact on the structure and dynamics of the nutrient network (44, 45). By their ability to produce extracellular enzymes and catalyze chemical alteration of detritus, the main ecological function of Labyrinthulomycetes may be that of saprotrophic decomposers (46). However, the molecular mechanism of their degradation of organic matter has not been fully studied. Two high-quality assembled genomes of cellulase-producing thraustochytrid strains reported in this study provide unprecedented evidence for the molecular pathway of carbon source utilization in an interesting yet understudied marine protist.

Thraustochytrids are members of Labyrinthulomycetes, which together with the photosynthetic Ochrophytes and the nonphotosynthetic Bicoeceans and Oomycetes belong to Stramenopiles (38). However, they display distinctly different nutrient uptake models. Labyrinthulomycetes have been recognized to be independent of true fungi but have evolved fungus-like patterns of osmotrophic nutrition (46). Moreover, studies have shown that the cells of Labyrinthulomycetes secrete an ectoplasmic network (15), which appears to help cells adhere to and penetrate the matrix and secrete digestive enzymes to dissolve nutrients for absorption (46). Perhaps, the ectoplasmic network supports the osmotic mode of nutrition in certain Labyrinthulomycetes, such as thraustochytrids.

Labyrinthulomycetes comprise three well-established groups, namely, labyrinthulids, aplanochytrids, and thraustochytrids (47). Labyrinthulids are usually parasites, symbionts, or mutualists that are prevalent on/in live marine algae and seagrasses. In contrast, thraustochytrids were rarely found on these living plants and they often thrive on dead native or exotic plant material, such as macroalgae and submerged mangrove leaves (18–21, 46). In our study, we identified several CAZymes in the genomes and secretomes of Labyrinthulomycetes strains, which are perhaps involved in the biodegradation of organic materials.

Cellulases play a key role in the degradation of plant debris. Through the cellulase assay, we confirmed the cellulase activities of the S-28 and S-429 strains (Fig. 2). Although the cellulase activities of these strains were comparatively lower than those reported in studies of other marine-derived cellulases (48–52), their quantitative detection provides the evidence for their potential to use plant-derived organic matter (wheat bran) as their carbon source. Furthermore, this study revealed that S-28 and S-429 genomes contain functional domains of the GH family. The GH family is a large enzyme group of glycoside hydrolases involved in the processing of carbohydrates and hydrolyzes glycosidic bonds in a variety of sugars (e.g., monosaccharides, oligosaccharides, polysaccharides, and glycoproteins) in living organisms (53). The key cellulases of the GH family (GH3, GH5_2, and GH9) were found in the secretomes of S-28 and S-429. All of the enzymes identified in our study are important for the decomposition of plant and animal detritus in marine environments. Therefore, our results support the earlier notion that thraustochytrids utilize highly refractory organic matter as a nutrient source by secreting extracellular enzymes (46).

This study found that some strains (e.g., SW8 and Mn4) lack CAZyme functional domains (Table 2), resulting in their inability to degrade certain carbohydrates independently. This was evident when these strains did not grow well on wheat bran as the sole carbon source (data not shown). Previous studies also reported that these strains do not grow well in culture medium containing only starch or carboxymethyl cellulose (CMC)-Na as the sole carbon source (29). Furthermore, it has been reported that 14 strains belonging to the *Aplanochytrium*, *Botryochytrium*, *Oblongichytrium*, *Parietichytrium*, *Schizochytrium*, *Sicyoidochytrium*, *Thraustochytrium*, and *Ulkenia* genera could produce cellulase when carboxymethyl cellulose was used as a substrate (16). In contrast, cellulolytic activity was not detected in any strains belonging to the *Aurantiochytrium* genus. These differences in the cellulose degradation ability of Labyrinthulomycetes seemingly reflect their contrasting niches and ecological roles in the marine ecosystem. Interestingly, many types of marine microorganisms have been confirmed to have the ability to produce cellulases, such as bacteria, fungi, yeast, and algae (54, 55), which is important for the purification of the marine environment and material circulation. Perhaps, cellulases broaden the niches of Labyrinthulomycetes and provide them with a powerful tool for competition. Furthermore, marine cellulase extracts have wide industrial and biotechnological applications, such as in the pharmaceutical industries, food, fuel, agriculture, and single-cell protein and as probiotics in aquaculture (55, 56). Our findings suggest that Labyrinthulomycetes can be an alternative microbial source of cellulases for industrial and biotechnological applications.

Besides being recognized as a vital decomposer in coastal environments (22), certain Labyrinthulomycetes are widely utilized in microbial fermentation for the production of less expensive yet valuable chemicals, such as docosahexaenoic acid (DHA) and squalene (57). The ability of Labyrinthulomycetes to utilize cellulose gives us the prospect to replace glucose with refractory organic matter such as leaves and wheat straw for lipid fermentation. Interestingly, cellulose-rich materials have been used as a substrate for the sustainable production of DHA and squalene (58–60). Furthermore, compared to terrestrial ecosystems, marine ecosystems have more potential to search for novel and stable cellulolytic enzymes because they have extreme temperature, salinity, and pressure conditions (61). Therefore, the cellulases discovered in Labyrinthulomycetes in our study can enrich the inventory of high-quality cellulolytic enzymes.

Cellulases are a group of enzymes consisting of endoglucanase, exoglucanase, and β-glucosidase, which play a synergistic role in the microbial degradation of cellulose (62, 63). At the genome level, we only found endoglucanase and β-glucosidase from the encoded proteins of Labyrinthulomycetes. However, when wheat bran was used as the substrate, the FPase, endoglucanase, and β-glucosidase activities were detected in S-28 and S-429 culture broths. Similar observations were noted in a previous study in which exoglucanase was not detected in the culture broth of a cellulose-degrading bacterium, *Serratia* sp. strain AXJ-M, but the strain was still able to degrade cellulose filter paper (64). Our findings suggest the possibility of a novel enzyme performing the exoglucanase activity in these Labyrinthulomycete strains.

A previous report has shown that endoglucanases can exhibit both endo- and exocellulase activities (65). When functioning as an exocellulase, it cleaves cellulose chains into cellotetraose units (66, 67). In our study, endoglucanases and exo-/endoglucanase were found to have similar protein structures and orientations of key residues. ThCel5A has been reported to be active against both amorphous cellulose and crystalline cellulose (68). Our experimental results also confirm that some Labyrinthulomycete strains could degrade cellulose independently. Taken together, we hypothesize that in cellulolytic Labyrinthulomycetes, many cellulases on the cell surface cooperate to complete the breakdown of cellulose. Our findings also suggest that endoglucanases of certain Labyrinthulomycetes may be novel exo-/endoglucanase and instigate future research on this topic. Interestingly, the morphological and functional characterization of these enzymes, as well as the uncovering of relevant genes, may become research hot spots. Besides, the exo-/endo-function of endoglucanases can be an excellent feature for metabolic engineering, which means that cells can obtain dual functions through the heterologous expression of a single enzyme. Thus, through the expression of endoglucanase in DHA-producing cells, the utilization of cellulose and the production of valuable products can be easily integrated.

Like bacteria, Labyrinthulomycetes can convert dissolved organic matter (DOM) into living biomass (69). Although they follow a seasonal pattern, Labyrinthulomycetes are found to match or even exceed bacterial biomass in the water column (9). They may feed on the leftover nutrients left behind by the bacteria, which is consistent with the current “leftover scavenger” nutritional model (9). In our study, we found that some species of Labyrinthulomycetes (e.g., *Aurantiochytrium*, as represented by strains SW8 and Mn4) are not able to produce cellulase for the breakdown of the plant cell wall and they may not be the first species to compete with bacteria. However, they do secrete other hydrolases that break down cellobiose into monosaccharides, which may help them digest nutrients left behind by the bacteria, thereby acting as leftover scavengers. Species from this group were found to produce DHA (29), which is a key essential fatty acid in the growth and maturation of higher-level trophic plankton. Thus, they may be able to play a more important role in the food web than their decomposing counterparts.

## MATERIALS AND METHODS

### Strains.

Two thraustochytrid strains, *Botryochytrium* sp. strain S-28 and *Oblongichytrium* sp. strain S-429, isolated from the coastal waters of Qingdao, China, were selected for whole-genome sequencing. The strains were previously identified by PCR amplification and sequence analysis of their full-length 18S rRNA genes (70). The isolates were maintained on modified Vishniac’s (MV) agar medium (glucose, 10 g/L; peptones, 1.5 g/L; yeast extract, 0.1 g/L; agar, 20 g/L; and artificial seawater, 33 g/L) at 28°C.

### Genome sequencing and assembly.

The strains S-28 and S-429 were cultivated at 28°C with reciprocal shaking (170 rpm) for 4 days. The culture medium (M4) contained glucose (20 g/L), peptones (1.5 g/L), yeast extract (1 g/L), KH_2_PO_4_ (0.25 g/L), and artificial seawater (33 g/L). Then, cells were harvested from 200 mL of fresh culture and genomic DNA was extracted using the cetyltrimethylammonium bromide (CTAB) method (71). The amount and quality of DNA were verified by NanoDrop One spectrophotometer (NanoDrop Technologies, Wilmington, DE), Qubit 3.0 fluorometer (Life Technologies, Carlsbad, CA, USA), and agarose gel electrophoresis at a commercial company (Lianchuan Biotechnology Co., Ltd., Hangzhou, China).

To obtain more accurate and in-depth data mining results, whole-genome sequencing of S-28 and S-429 was performed on the Illumina NovaSeq and Oxford Nanopore PromethION sequencing platforms. The Guppy toolkit (https://nanoporetech.com/community) was used for base calling and filtering of low-quality Oxford Nanopore long reads. The Fastp tool was used for quality assessment and data filtration (72). The raw data were filtered to (i) remove reads with an N base content of >5%, (ii) remove reads with low-quality bases reaching 50% (quality [Q] score of ≤5), (iii) remove reads with adapter contamination, (iv) remove reads with PCR duplicates, and (v) remove reads with an average quality value (Q score) of ≤7 to obtain clean sequencing data. The Q score is an important index to measure the quality of sequencing. A higher-quality value represents the smaller probability of wrong sequencing. The second generation and the third generation filtered out the low-quality sequences based on Q scores of ≤5 and ≤7, respectively. The k-mer-based analysis method was used to estimate the genome size and heterozygosity (73). After genome error correction, a hybrid Illumina + Nanopore assembly was constructed by NextDenovo (version 2.3.1) for S-28 and NECAT (version 20200119) for S-429. Racon (version 1.4.13) was used to perform error correction using the Nanopore sequencing data. Pilon (version 1.23) (74) was used for further error correction of the assembly with Illumina reads. Finally, after heterozygosity removal, the assembly statistics were obtained. Genome completeness was assessed by BUSCO (version 4.1.4) using a eukaryotic model (37). Genome sequencing and assembly were performed at Lianchuan Biotechnology Co., Ltd. (Hangzhou, China).

### Gene prediction and functional annotation.

Protein-coding genes were predicted using BRAKER (version 2.1.5) leveraging the Augustus and GeneMark-ES tools (75). DIAMOND (version 2.0.4) was used to obtain the functional information of sequences and the metabolic pathways (76). HMMER (version 3.2.1) was used to predict the domain and obtain the conserved sequence, motif, and domain of the protein (77). The expect values (E values) for DIAMOND and HMMER were 1e−5 and 0.01, respectively. Genes were annotated using the Non-Redundant Protein Sequence Database (NR), Swiss-Prot, Cluster of Orthologous Groups of proteins (COG), Kyoto Encyclopedia of Genes and Genomes (KEGG), and Gene Ontology (GO) databases. The predicted proteins were also functionally annotated by InterProScan to specify GO terms and InterPro (IPR) domains. The enrichment of IPR domains classified as hydrolase activity in GO annotations was analyzed using the hypergeometric distribution implemented in the R package heatmap. The significance values were adjusted for multiple testing using the *q* value (set to 5% allowable false-discovery rate [FDR]). The enzymes related to carbon metabolism were annotated according to the CAZymes database using the dbCAN2 (https://bcb.unl.edu/dbCAN2/index.php) meta server with HMMER search against the dbCAN HMM (hidden Markov model) database (78). To identify annotated CAZymes belonging to the secretomes of thraustochytrids, the predicted proteins of CAZymes were analyzed using the SignalP 5.0 server (https://services.healthtech.dtu.dk/service.php?SignalP-5.0) (79). Additionally, to understand functional genic information among thraustochytrid strains, annotation of orthologous gene clusters was carried out by OrthoVenn2 (https://orthovenn2.bioinfotoolkits.net/home) (80). Gene prediction and annotation were performed at Lianchuan Biotechnology Co., Ltd. (Hangzhou, China).

### Public data for comparative analysis.

Genome assemblies and annotations of 19 other microorganisms were used in this study. These included five Oomycetes species, five Ochrophyta species, four Fungi species, and five Labyrinthulomycetes species. Additional genome assemblies and annotations were retrieved from the Joint Genome Institute (JGI) database (81). The accession numbers of these 19 other microorganisms are listed in Table S7 in the supplemental material.

### Phylogenetic analysis.

Phylogenetic relationships of 26 Labyrinthulomycete strains were inferred by analysis of 18S rRNA sequences retrieved from the NCBI database. A maximum likelihood (ML) tree was constructed using MEGA7.0, and clade relationships and tree topology confidence were assessed with 1,000 bootstrap replicates (82).

To infer relationships between the genomes of seven Labyrinthulomycetes and 14 additional complete genomes, a collection of single-copy orthologous gene families was identified with OrthoFinder (83). The 121 single-copy gene families found in all 21 species were selected from the clustering, and the protein sequences were aligned using MUSCLE 3.8.31 (84). ProtTest (85) was used to predict suitable amino acid substitution models for the construction of a phylogenetic tree. These alignments were concatenated, and an ML tree was computed with RAxML (86) using the PROTGAMMALGX amino acid substitution model (determined as the best model by ProTest) and 1,000 bootstrap replicates.

### Protein structure prediction of annotated cellulases.

To obtain conserved regions of proteins, multiple-sequence alignment (MSA) was first performed using ClustalX 1.83 (87). The three-dimensional structure of predicted cellulases was constructed by the SWISS-MODEL workspace (88) via the selection of the most similar template. The MSA and predicted protein structure file were linked to the ConSurf server (89) for rendering of protein structure. Each residue was scored as per its conservational status in ConSurf. Then, the UCSF Chimera tool (90) was used to visualize the three-dimensional protein structure of cellulase. Endo/exocellulase E4 (E4) belonging to family 9 of glycoside hydrolases (GH9) from Thermomonospora fusca (43) and a GH5 exo/endoglucanase, ThCel5A (ThCel5A), of Thermobifida halotolerans YIM 90462^T^ (68) were selected as reference cellulases for structure and active site comparison.

### Cellulase assay.

Qualitative screening of cellulolytic activities of the S-28, S-429, and Mn4 strains was performed by inoculating their colonies onto Congo red plates (CMC-Na, 2 g/L; NH_4_Cl, 1 g/L; MgSO_4_·7H_2_O, 0.05 g/L; Congo red, 0.4 g/L; K_2_HPO_4_, 0.05 g/L; agar, 16 g/L) followed by the observation of clearance zones. The activities of cellulases (i.e., FPase, CMCase, and β-glucosidase) were further quantified by the dinitrosalicylic acid (DNS) method (91). Filter paper and 1% CMC-Na were used as the substrates, respectively. The strains were first cultured in an M4 medium (92) at 28°C until the logarithmic growth phase to obtain seed cultures for quantification of cellulase activity. The seed culture (3 mL) was centrifuged at 4,000 rpm at 25°C for 10 min. The supernatant was discarded, and the cell pellet was washed twice with sterilized artificial seawater (33 g/L). Thereafter, the washed pellet was suspended in the wheat bran fermentation medium [wheat bran, 30 g/L; (NH_4_)_2_SO_4_, 5 g/L; MgSO_4_·7H_2_O, 0.5 g/L; NaCl, 0.5 g/L; K_2_HPO_4_, 0.5 g/L] and cultivated at 28°C and 170 rpm for 15 days. The culture was centrifuged at 8,000 rpm and 4°C for 10 min, and the resulting supernatant was used for the determination of cellulase activities. The release of reducing sugars was measured at 540 nm, and the enzyme unit (units per milliliter) was defined as the amount of enzyme that produces 1 μmol of reducing sugar per mL per min (93). The β-glucosidase activity was determined by using *p*-nitrophenol-d-glucopyranoside (pNPG) as the substrate. The release of *p*-nitrophenol was measured at 410 nm (94), and the enzyme unit (units per milliliter) of β-glucosidase activity was defined as the release of 1 μmol *p*-nitrophenol per mL per min (93).

### Data availability.

The data have been deposited with links to BioProject accession no. PRJNA844486 and PRJNA844489 in the NCBI BioProject database (https://www.ncbi.nlm.nih.gov/bioproject/). Data may be available from the corresponding author upon reasonable request.
