# Supplementary material for: Comparative Genomic Analyses of Cellulolytic Machinery Reveal Two Nutritional Strategies of Marine Labyrinthulomycetes Protists
Source: Microbiol Spectr. 2023 Feb 6;11(2):e04247-22. doi: 10.1128/spectrum.04247-22 (PMC10101102; doi:10.1128/spectrum.04247-22)
Supplement: Supplemental file 2 — Fig. S1 to S6. Download spectrum.04247-22-s0002.pdf, PDF file, 0.3 MB [file spectrum.04247-22-s0002.pdf]

Supplementary Figures

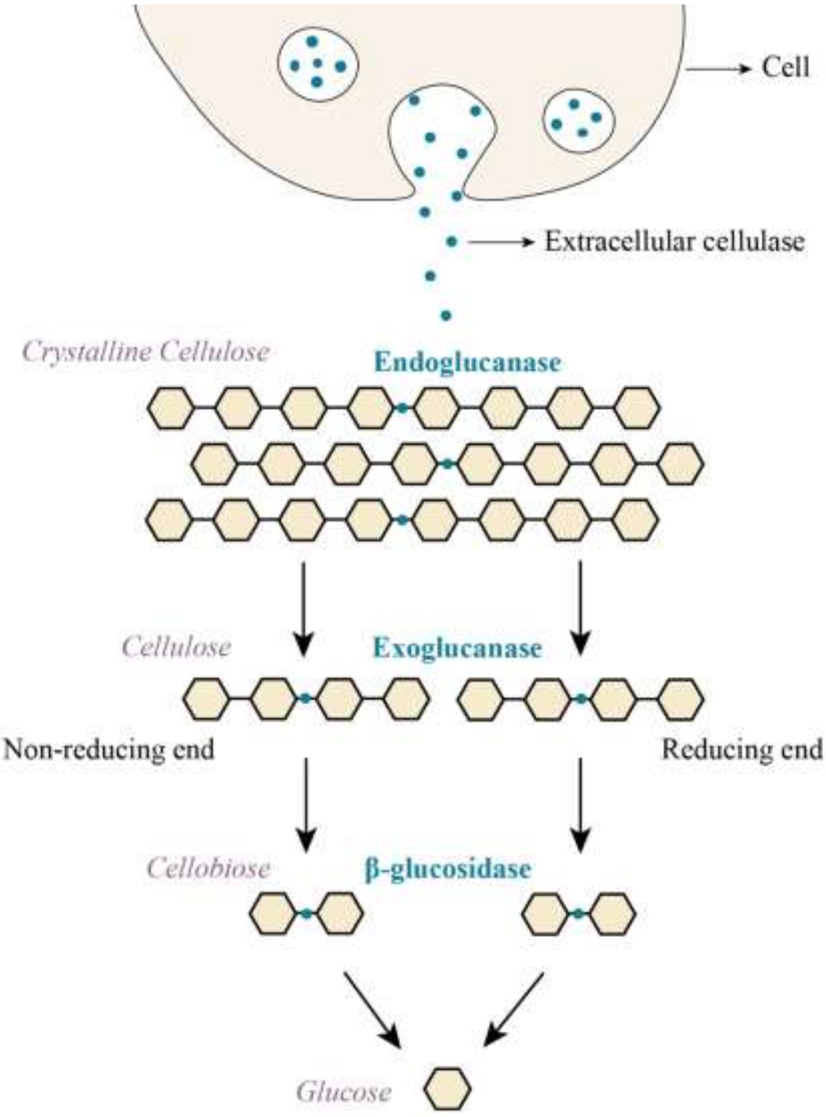

Fig. S1 Breakdown of crystalline cellulose by microorganisms [1].

Reference

1. Arora, M., R.M. Yennamalli, and T.Z. Sen, *Application of Molecular Simulations Toward Understanding Cellulase Mechanisms.* BioEnergy Research, 2018. **11**(4): p. 850-867.

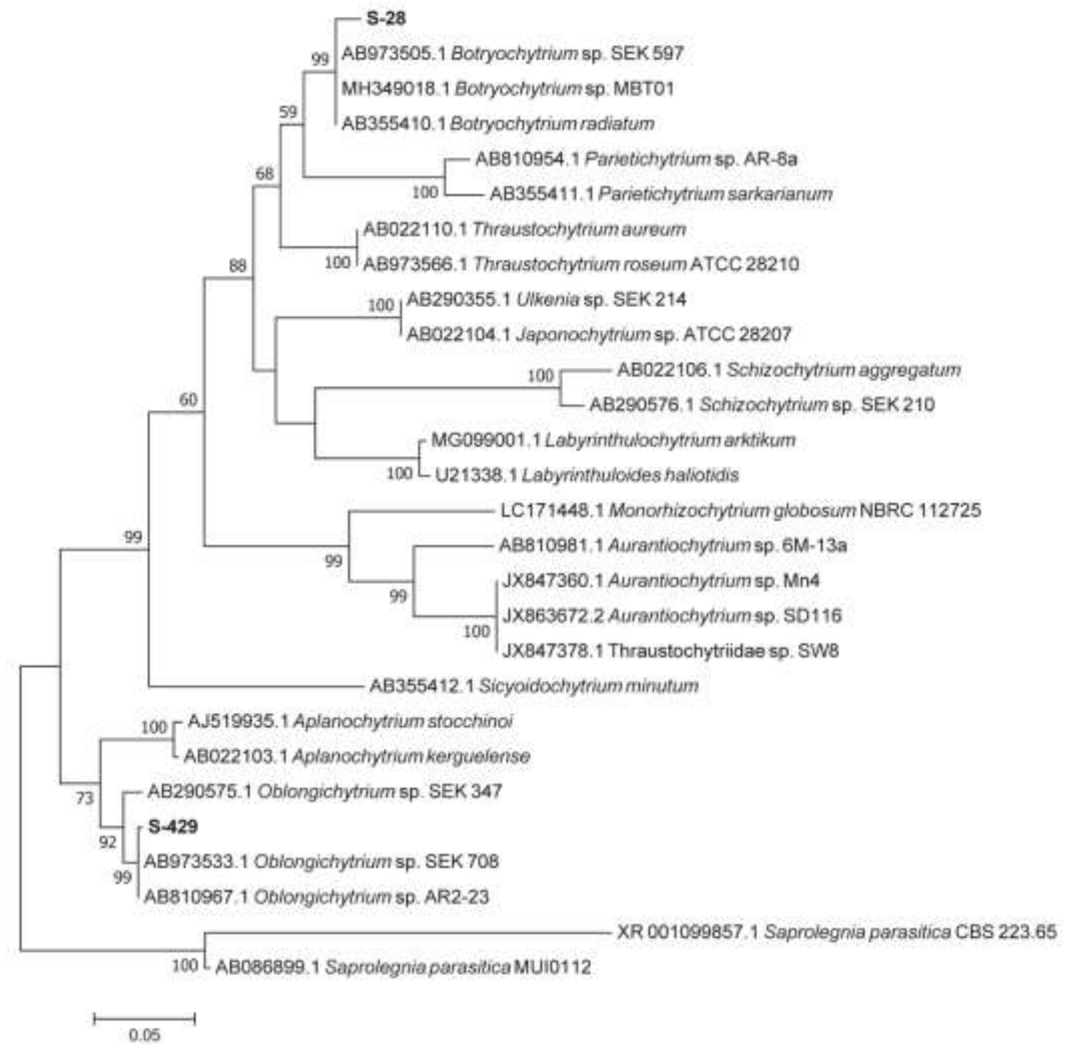

Fig. S2 Phylogenetic tree of thraustochytrids using maximum-likelihood method based on 18S rRNA gene sequences.

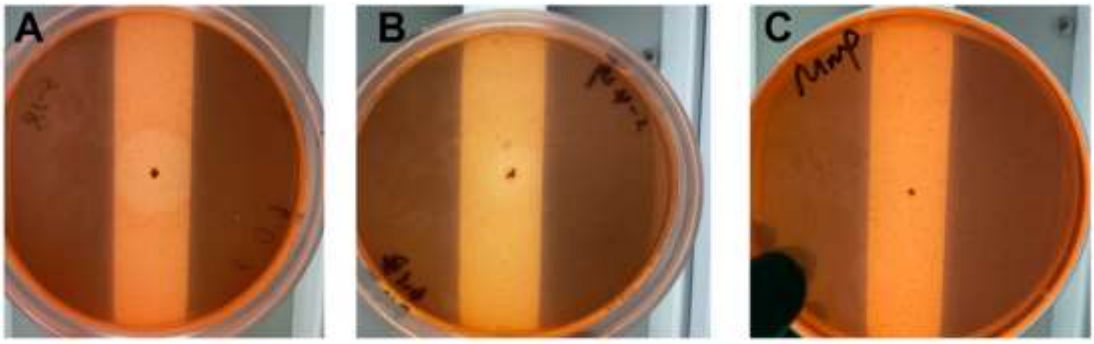

Fig. S3 Transparent circles on plates inoculated with (**A**) S-28, (**B**) S-429, and (**C**) Mn4 strains.

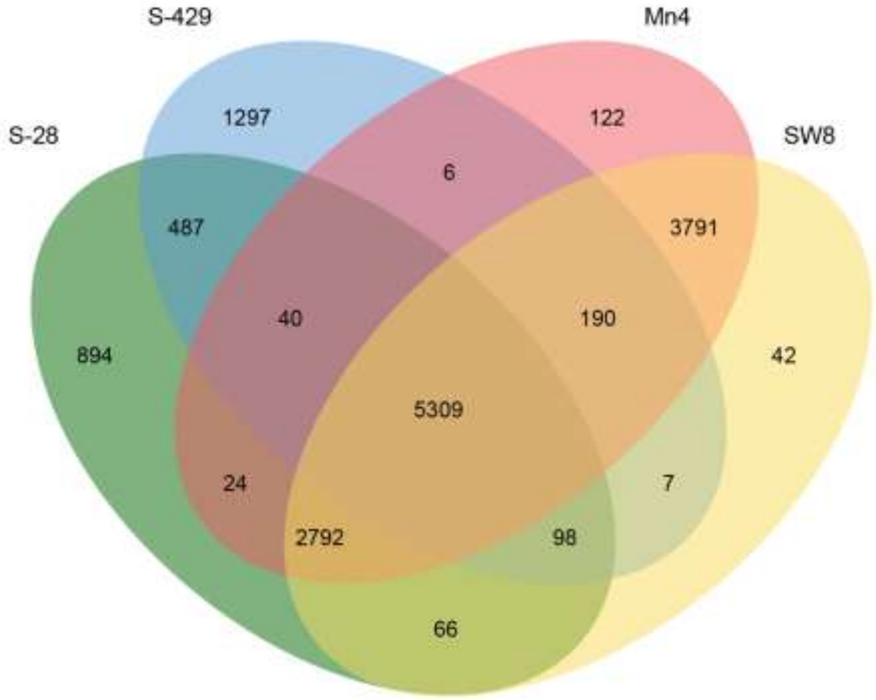

Fig. S4 Venn diagram representing distribution of shared and unique orthologous groups between cellulase-producing (S-28 and S-429) and non-cellulase producing strains (Mn4 and SW8).

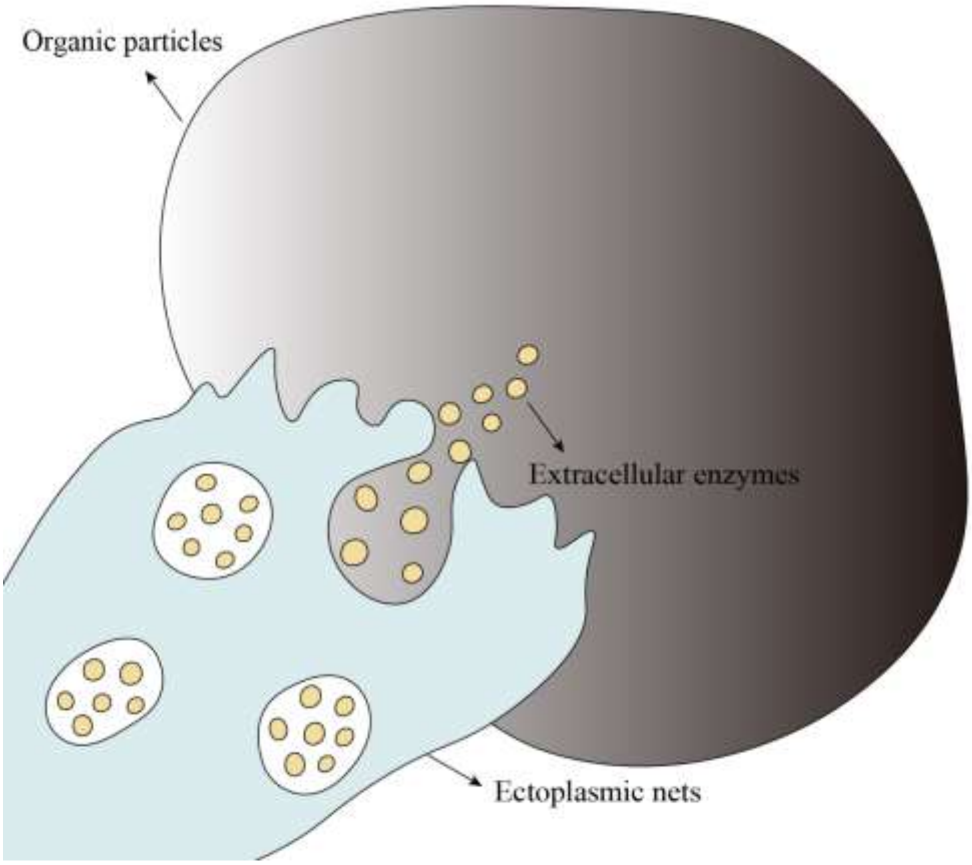

Fig. S5 A hypothetical process by which thraustochytrids secrete extracellular enzymes through endoplasmic nets to penetrate organic particles.

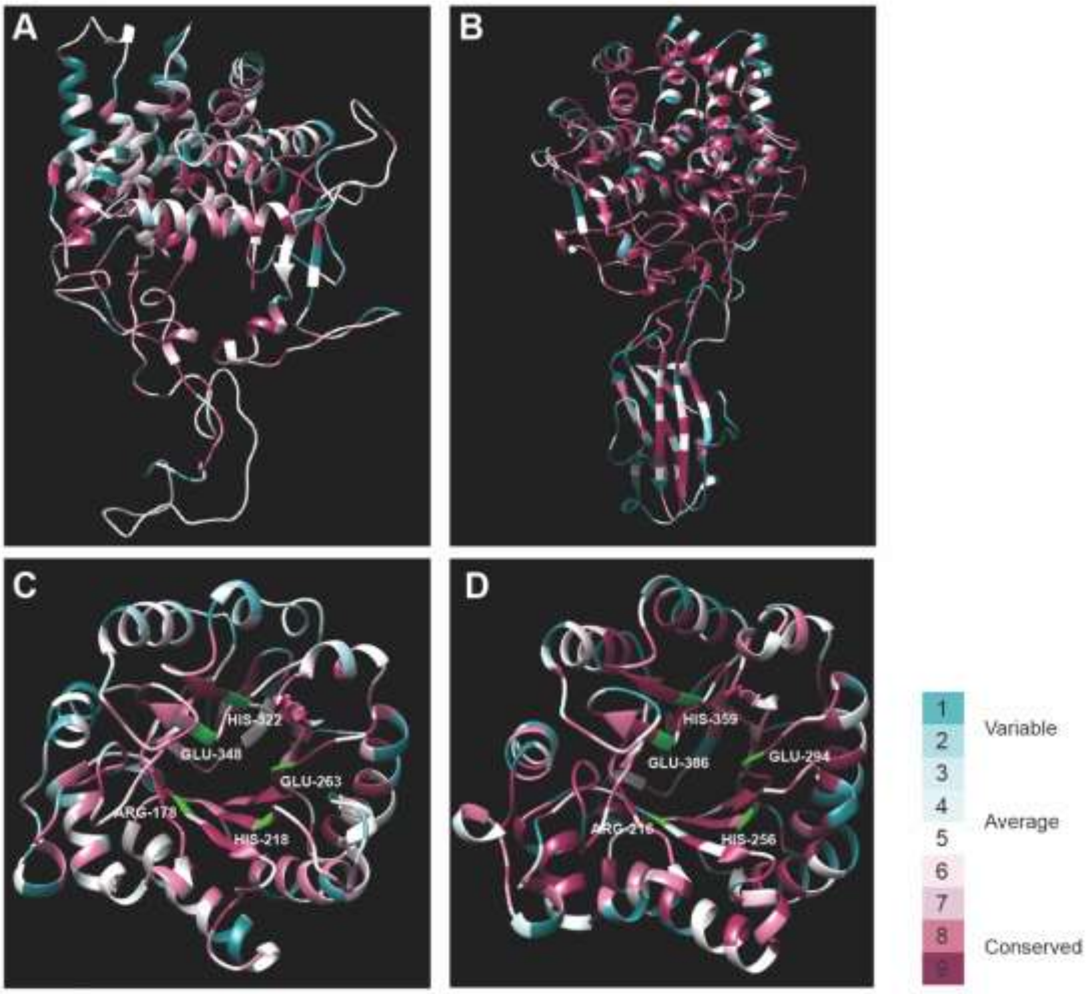

Fig. S6 Comparison of predicted three-dimensional protein structure of endoglucanase ThrCel9 (**A**) and E4 (**B**) from GH9 family, and endoglucanase ThrCel5 (**C**) and ThCel5A (**D**) from GH5 family. The conservation status of rotatable molecule was colored along a color scale of green (low) to red (high) according to ConSurf conservation scores.
